# Prediction of VEGF-C as a Key Target of Pure Total Flavonoids From Citrus Against NAFLD in Mice via Network Pharmacology

**DOI:** 10.3389/fphar.2019.00582

**Published:** 2019-06-04

**Authors:** Wei Hong, Songsong Li, Liyan Wu, Beihui He, Jianping Jiang, Zhiyun Chen

**Affiliations:** ^1^The Second Central Laboratory, The First Affiliated Hospital of Zhejiang Chinese Medical University, Hangzhou, China; ^2^Key Laboratory of Integrative Chinese and Western Medicine for the Diagnosis and Treatment of Circulatory Diseases of Zhejiang Province, Hangzhou, China

**Keywords:** pure total flavonoids from citrus, non-alcoholic fatty liver disease, vascular endothelial growth factor-C, network pharmacology, non-alcoholic steatohepatitis

## Abstract

Pure total flavonoids from Citrus (PTFC) effectively reduce the symptoms of non-alcoholic fatty liver disease (NAFLD). Our previous microarray analysis uncovered the alterations of important signaling pathways in the treatment of NAFLD with PTFC. However, the underlying core genes that might be targeted by PTFC, which play important roles in the progression of NALFD are yet to be identified. In this study, we predicted the vascular endothelial growth factor-C (VEGF-C) as potential key molecular target of PTFC against NAFLD via network pharmacology analysis. The network pharmacology approach presented here provided important clues for understanding the mechanisms of PTFC treatment in the development of NAFLD.

## Introduction

Non-alcoholic fatty liver disease (NAFLD) is rapidly becoming a major healthcare problem worldwide affecting 15–30% population in Asia (Srivastava et al., 2017). It is defined as abnormal hepatic lipid accumulation (>5% by weight) without excessive alcohol intake (Nalbantoglu and Brunt, 2014). NAFLD is considered to be a hepatic manifestation of metabolic syndrome (Takahashi et al., 2015), which is closely associated with obesity, insulin resistance, diabetes and hypertriglyceridemia (Kirpich et al., 2011). NAFLD may progress from simple steatosis (SS) into a more severe form, non-alcoholic steatohepatitis (NASH) (Konerman et al., 2017). NASH is typically characterized by ballooning degeneration, inflammation and fibrosis. It may lead to cirrhosis and hepatocellular carcinoma (HCC) without intervention or treatment (Miele et al., 2007; Chow et al., 2017; Konerman et al., 2017). Recently, NASH has become the third most common indication for liver transplantation in the United States (Takahashi et al., 2015).

To date, the underlying mechanism of NAFLD progression is largely unknown and there is no established pharmacotherapy for NAFLD except for life style modification by diet and exercise (Takahashi et al., 2015). In recent years, growing attention has been paid to natural products or Chinese herbal medicine intervention as a promising alternative for the treatment of NAFLD (Takahashi et al., 2015; Xu et al., 2015; Chen et al., 2017). We previously found that pure total flavonoids from Citrus (PTFC) attenuated NASH symptoms. Naringin, neohesperidin and narirutin are three major components of PTFC and the total flavonoid content exceeds 75% (Wu et al., 2017). Naringin possesses diverse pharmacological properties including anti-inflammation, against oxidative stress and apoptosis (Chen et al., 2016). Neohesperidin functions in inactivating nuclear factor kappa B (NF-κB) involved inflammation pathway and suppressing nuclear factor of activated T-cells (NFAT) and calcium oscillations (Tan et al., 2017). In addition, neohesperidin also has hypoglycemic and hypolipidemic effects (Jia et al., 2015). Narirutin has been reported to prevent lipid formation and suppress inflammation as well as antioxidation (Chakraborty and Basu, 2017). Anti-inflammation (Wu et al., 2017) and antioxidation (Jiang et al., 2014) play important roles in PTFC treatment. However, limited information is available about the relationship between the progression of NAFLD and PTFC treatment. The underlying core genes that might be targeted by PTFC, which play important roles in the progression of NALFD are not yet clear.

Network pharmacology (Hopkins, 2008) is based on the principles of network theory and systems biology, which explores the link between drugs and disease from a holistic perspective and is coincided with the characteristics of multi-component, multi-target and multi-pathway of Chinese herbal medicine. In recent years, network pharmacology combined with high-throughput omics detection has been increasingly widely used in the target prediction of drugs, active components identification and/or pharmacological mechanisms analysis of natural products or traditional Chinese medicine (Liu et al., 2015; Gu and Pei, 2017; Isgut et al., 2017). In this study, we constructed the networks of NAFLD progression and PTFC treatment in parallel via network pharmacology analysis to find the common gene targets as the potential molecular targets of PTFC with the previous raw data we obtained.

The previous microarray data which included microarray information of C57BL/6 mice fed with high-fat diet (HFD) for different time or intervened with PTFC was categorized into NAFLD progression group and PTFC treatment group, and then was analyzed in parallel to find the common target nodes of the networks between these two groups. Out of our expectation, vascular endothelial growth factor-C (VEGF-C), the crucial regulator of lymphangiogenesis was identified as the key potential target of PTFC against NAFLD. Our new finding indicated that the dynamic changes in the expression of VEGF-C may play important roles in the progression of NAFLD and targeting for VEGF-C might be one of the main mechanisms of PTFC treatment.

## Materials and Methods

### The Source of Microarray Data

In our previous study (Wu et al., 2017), we reported the alteration of TLR/CCL signaling pathway among ND (normal diet) group, 24-week HFD fed group and PTFC treatment group, in which the data of 16-week HFD fed group were not included. In the present study, we used the microarray data of ND, HFD for 16- and 24-week groups combined with PTFC treatment group for our follow-up analysis. As the report described, control group SPF C57BL/6 mice were fed with ND for 24-week, HFD group mice were fed with HFD for 16 and 24-week. Mice with HFD 6-week received intragastric administration with PTFC for 18-week. The Mouse OneArray@v2 gene chip was used to measure the gene expression profiles. The raw microarray data were uploaded to the Gene Expression Omnibus (GEO) database^1^.

### The Whole Workflow of Network Pharmacology Strategy

**FIGURE 1:**
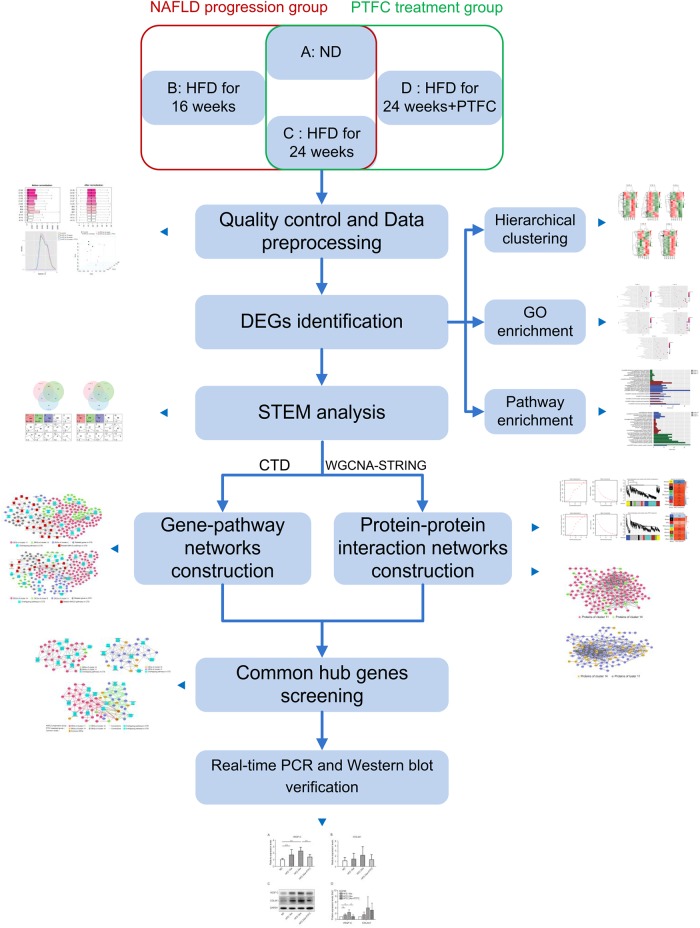
The whole workflow of network pharmacology strategy to screen common target nodes for NAFLD progression group and PTFC treatment group. ND, normal diet; HFD, high-fat diet; DEG, differentially expressed gene; STEM, short time-series expression miner; CTD, the comparative toxicogenomics database; WGCNA, weighted gene co-expression network analysis; SRING: string database.

The workflow of this study is summarized in Figure 1. We firstly categorized the microarray data into NAFLD progression group (subgroups A, ND for 24-week; B, HFD for 16-week; C, HFD for 24-week) and PTFC treatment group (subgroups A, ND for 24-week; C, HFD for 24-week; D, HFD for 24-week combined with PTFC intervention for 18-week). After quality control and data preprocessing, the differentially expressed genes (DEGs) were identified. Meanwhile, the gene ontology (GO) and the Kyoto encyclopedia of genes and genomes (KEGG) pathway enrichment analyses were performed. The DEGs in each group were clustered by using Short Time-series Expression Miner (STEM). The gene-pathway networks were constructed based on the CTD^2^. To refine the genes that were significantly associated with NAFLD progression or PTFC treatment, we performed weighted gene co-expression network analysis (WGCNA) on the DEGs obtained via STEM. The protein-protein interactions were subsequently identified using the STRING database^3^. Integrating the analyses based on CTD and WGCNA-STRING, gene-pathway networks of NAFLD progression and PTFC treatment groups were established to identify the common genes and pathways that play major regulatory roles in the progression of NALFD and PTFC treatment.

### Data Quality Control and Preprocessing

The raw data (GPR files from Agilent standard array) were transferred to a recognizable expression profiling format by using Express Converter Version 2.1 (Sioson et al., 2006)^4^ and the full matrix of gene expression was obtained. Limma package (Version 3.32.5) of R (3.4.1)^5^ was used to normalize the gene expression profiling. Density distribution of the normalized gene expression profiles was performed by using ggplot2 package of R (3.4.1). The Principal components analysis was performed by using Psych package Version 1.7.8 of R(3.4.1)^6^ (Condon and Revelle, 2014).

### DEGs Identification

The DEGs within groups B-A, C-A, D-A, C-B and D-C were identified by Limma (Version 3.32.5) of R (3.4.1) (see text footnote 5) package (*P* < 0.05 and |logFC|>1. Pheatmap package (Version 1.0.8) (Wang et al., 2014)^7^ was applied to perform hierarchical clustering.

### GO Biological Process and KEGG Pathway Enrichment

GO Biological Process and KEGG pathway enrichment were conducted using DAVID version 6.8^8^ (Huang et al., 2009a,b).We used Cytoscape3.5.1^9^ (Shannon et al., 2003) to visualize the pathways and the related genes.

**Table 1:** The number of DEGs up- or down-regulated in each pairwise comparisons.

Comparisons	Down-regulated DEGs	Up-regulated DEGs	Total DEGs
B vs. A	368	1004	1372
C vs. A	275	1178	1453
D vs. A	126	293	419
C vs. B	33	57	90
D vs. C	544	121	665

*A, ND for 24-week; B, HFD for 16-week; C, HFD for 24-week; D, HFD for 24-week combined with PTFC intervention for 18-week.*

### STEM Clustering Analysis

Venn diagrams were generated using Venn Diagram (Version 1.6.17)^10^ of R (3.4.1) (Chen and Boutros, 2011). Clustering analysis of DEGs was carried out using short time-series expression miner (STEM) (version1.3.11)^11^ (Shannon et al., 2003). The correlation coefficient of gene expression in each cluster was set higher than 0.8 and the significance *p*-value was less than 0.05.

### Network Construction Based on CTD

We first downloaded KEGG pathways and associated genes from CTD (see text footnote 2) with the key word “non-alcoholic fatty liver disease, NAFLD.” We compared these information with the DEGs clustered by using STEM and their associated pathways and constructed the networks of gene-pathway. Visualization was performed by using Cytoscape3.5.1 (see text footnote 9).

### WGCNA Analysis and Protein-Protein Interaction Network Construction

WGCNA analysis was performed by using WGCNA package (Version 1.61) of R (3.4.1) (Langfelder and Horvath, 2008)^12^. STRING Version 10.5 database (Szklarczyk et al., 2017)^13^ was applied to construct the network of protein-protein interaction. Visulization was performed by using Cytoscape3.5.1 (see text footnote 9).

### Real-Time PCR, Western Blot Assay and Statistical Analysis

Total RNAs from liver tissues of the 24 mice (*n* = 6/group) were extracted by using Takara MiniBEST Universal RNA Extraction Kit (Takara, Dalian, China; Cat # 9767). PrimeScript kit (Takara, Dalian, China; Cat # RR820A) was used to synthesize cDNA, according to the manufacturer’s method. real-time PCR was performed with C1000^TM^ Thermal Cycler CFX 384 (Bio-Rad) and SYBR Premix EX Taq (Takara, Dalian, China; Cat # RR420A) as previously described (Wu et al., 2017). Relative transcript levels were calculated via the 2^−ΔΔC(t)^ method and β-actin transcripts were used as the internal control. The primer sequences are shown as follows. VEGF-C: GTG AGG TGT GTA TAG ATG TGG GG (forward), ACG TCT TGC TGA GGT AAC CTG (reverse); COL4A1: CCT GGC ACA AAA GGG ACG A (forward), ACG TGG CCG AGA ATT TCA CC (reverse); CCL4: TTCCTGCTGTTTCTCTTACACCT (forward), CTGTCTGCCTCTTTTGGTCAG (reverse); CCR7: TCATTGCCGTGGTGGTAGTCTTCA (forward), ATGTTGAGCTGCTTGCTGGTTTCG (reverse). Western blot was performed as we previously described (Wu et al., 2017). The antibodies of VEGF-C (ab191274) and COL4A1 (ab135802) were purchased from Abcam Trading Co., Ltd., and were diluted 1:500 or 1:30. The antibodies of GAPDH (Mab5465-100) and Horseradish peroxidase-conjugated immuno-globulin G antibodies (GAM0072, GAR0072) were purchased from MultiSciences Biotech, Co., Ltd., and were diluted 1:5000. Blots were imaged and quantified using Odyssey Fc imaging system (LI-COR Biosciences).

Data were analyzed using SPSS17.0 software. One-way analysis of variance was used and results are reported as mean ± standard deviation. LSD analysis (homogeneity of variance) was used on comparison among groups. *P* < 0.05 was statistically significant.

## Results

### Data Preprocessing and DEGs Screening

**FIGURE 2:**
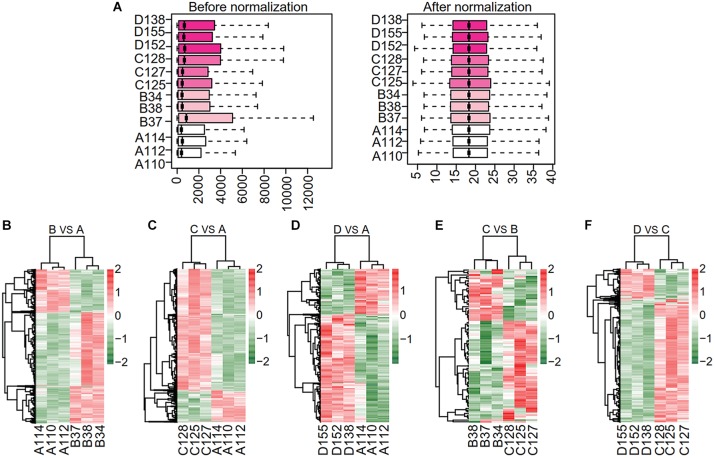
Data normalization and hierarchical clustering. **(A)** Box plots of normalized data for expression profile of all samples. Data distribution before (left) and after (right) normalization. The vertical axis represents all samples. **(B–F)** Hierarchical clustering analysis of differentially expressed gene among the samples. Each gene is represented in each row and the column denotes each run. Scale bar denotes the *Z*-score fold change. The vertical axis represents all samples among pairwise comparisons of subgroups B vs. A, C vs. A, D vs. A, C vs. B and D vs. C. A, ND for 24-week; B, HFD for 16-week; C, HFD for 24-week; D, HFD for 24-week combined with PTFC intervention for 18-week.

Raw microarray data were converted into a recognizable expression profiling format and the gene expression matrix was obtained. A total of 20,105 genes were detected. We normalized the expression matrix and 20,070 non-redundant genes were identified. The normalized expression data shown with box plots (Figure 2A) indicated the reliability of the data. The density distribution analysis showed that the density in subgroups A, B, C, and D had similar skewed distribution (see section“The Source of Microarray Data” Supplementary Figure S1A). The principal component analysis (PCA) plot showed a clear distribution of all samples (Supplementary Figure S1B).

We identified the DEGs in five pairwise comparisons: B vs. A, C vs. A, D vs. A, C vs. B and D vs. C using Limma package (*P* < 0.05 and |logFC| > 1. The up- or down-regulated DEGs in each comparison were summarized in Table 1. Bidirectional hierarchical clustering heatmap was generated according to the expression levels of DEGs in each comparison (Figure 2B–F and Supplementary Table S1). The DEGs in the above five comparisons were mainly involved in biology processes of immune response (GO:0006955), cell activation (GO:0001775), immune response (GO:0006955), regulation of cytokine production (GO:0001817) and immune response (GO:0006955) respectively. They mainly acted in Chemokine signaling pathway (mmu04062), Cytokine-cytokine Receptor Interaction pathway (mmu04060), Cytokine-cytokine Receptor Interaction pathway (mmu04060) and Chemokine signaling pathway (mmu04062) separately. (Supplementary Figure S2 and Supplementary Table S2).

### STEM Clustering of DEGs of NAFLD Processing Group and PFTC Treatment Group

**FIGURE 3:**
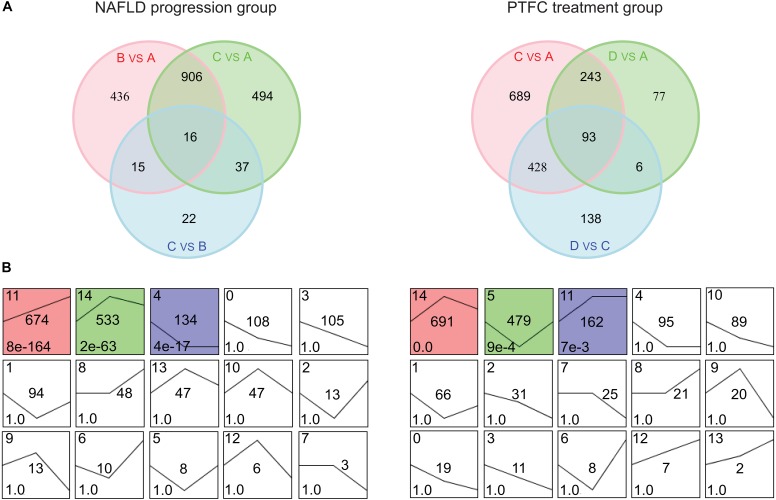
Venn diagrams and STEM clustering analysis of the DEGs in NAFLD progression group and PTFC treatment group. **(A)** Venn diagrams showing the number of DEGs in the three pairwise comparisons in NAFLD progression group (left) and PTFC treatment group (right). **(B)** STEM clustering of DEGs expression patterns of NAFLD progression group (left) and PTFC treatment group (right). Each box shows a clustering of gene expression pattern. The cluster ID number is marked in the top left-hand corner of the box and the curve denotes gene expression tendency in different sample subgroups. The number in the middle is the number of DEGs. The *P*-value of clustered time series genes is marked in the bottom left-hand corner of each cluster box. The colored box denotes significant clustering (*P* < 0.05).

Further analysis was performed based on two main lines: the line of NAFLD progression group (subgroups A, B, and C) and the line of PTFC treatment group (subgroups A, C, and D). We firstly analyzed the gene sets in the two groups. As shown in Figure 3A, the two collections of gene sets contained 1,926 and 1,674 DEGs, respectively (Supplementary Table S3). Next, the STEM analysis was performed with these 1,926 and 1,674 DEGs to analyze the gene expression patterns. Three significant clusters were identified from the combination gene sets of NAFLD progress group, including 674, 533 and 134 DEGs (Figure 3B left and Supplementary Table S4). For the PTFC treatment group, there were three gene clusters were identified, which included 691, 479, and 162 DEGs, respectively (Figure 3B right and Supplementary Table S4).

Kyoto encyclopedia of genes and genomes pathway enrichment analysis was conducted with these DEGs from the above clusters. The data showed that DEGs in cluster 11, 14, and 4 in NAFLD progression group mainly participated in Cytokine-cytokine Receptor Interaction pathway (mmu04060), Cell Adhesion Molecules pathway (mmu04514) and Steroid Biosynthesis pathway (mmu00100) et al. (Supplementary Figure S3A and Supplementary Table S5). And DEGs in cluster 14, 5, and 11 in PTFC treatment group mainly functioned in pathways of Chemokine Signaling (mmu04062), Biosynthesis of Unsaturated Fatty Acids (mmu01040) and Sphingolipid Metabolism (mmu00600) et al. (Supplementary Figure 3B and Supplementary Table S5).

**FIGURE 4:**
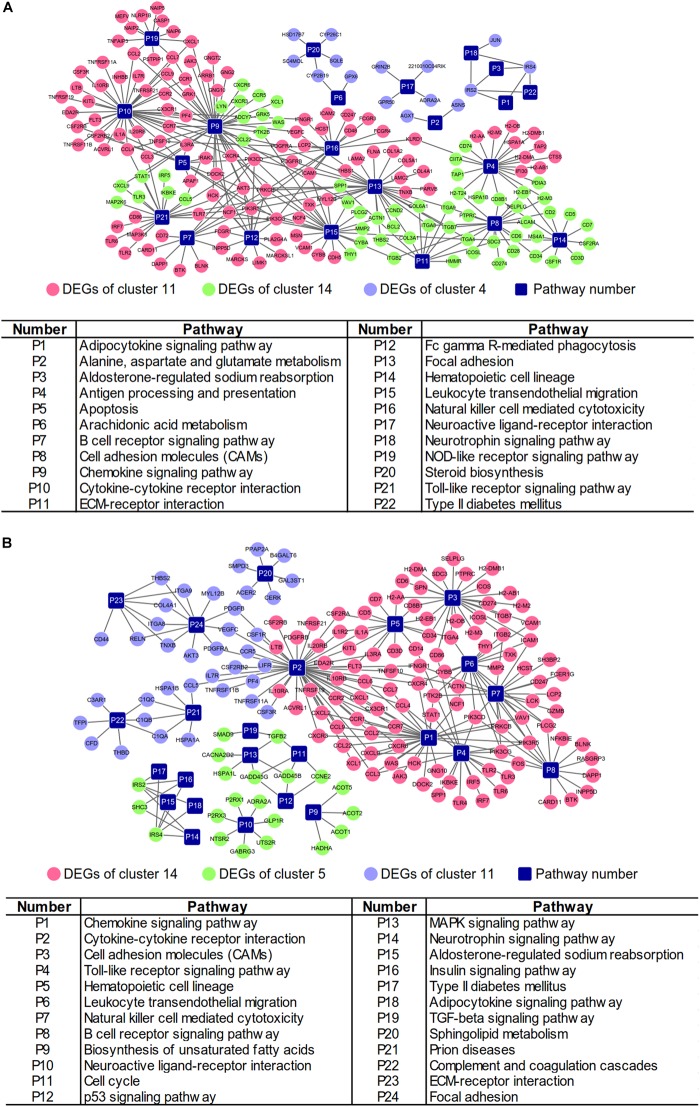
Gene-pathway networks construction. **(A)** Network of NAFLD progression group. **(B)** Network of PTFC treatment group.

Integrated with the above DEGs and associated pathways, the gene-pathway networks analysis was established. For the NAFLD progression group, 208 nodes were involved in the network including 22 pathways, 186 genes (111 DEGs from cluster 11, 60 DEGs from cluster 14 and 15 DEGs from cluster 4) and 292 connections (Figure 4A). The network of PTFC treatment contained 188 nodes, including 24 pathways, 106 genes (106 DEGs from cluster 14, 21 DEGs from cluster 5 and 37 DEGs from cluster 11) and 261 connections (Figure 4B).

### Networks Construction Based on CTD

**FIGURE 5:**
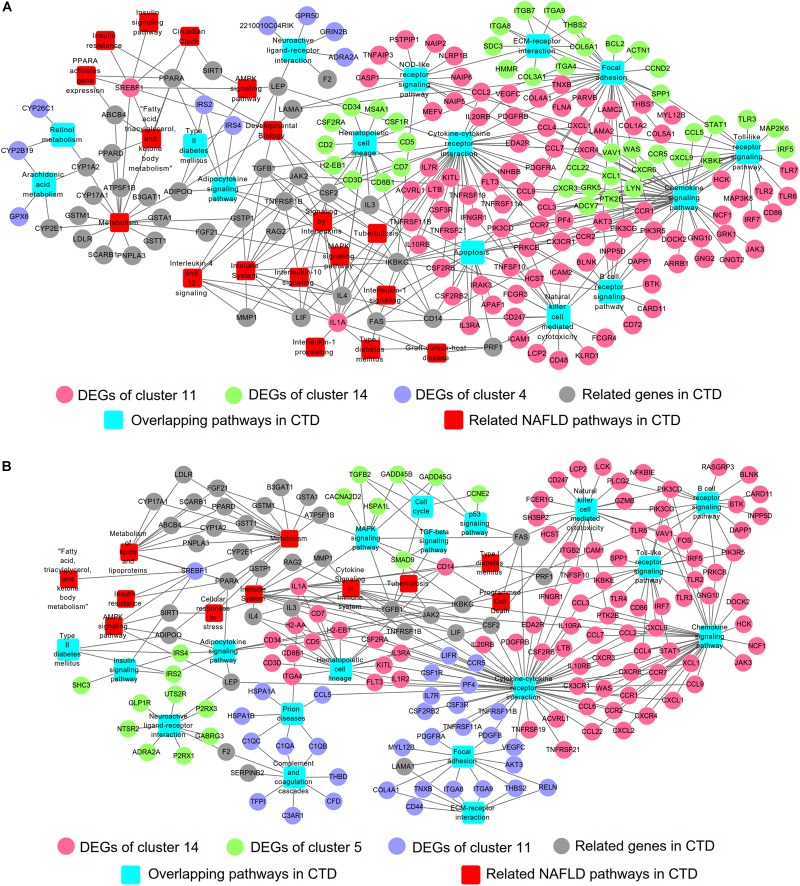
Gene-pathway network construction based on CTD. **(A)** Network of NAFLD progression group. **(B)** Network of PTFC treatment group.

The Comparative Toxicogenomics Database (CTD^14^) was initially developed to formalize the information of environmental toxic agent and gene products (Davis et al., 2015). After more than 10 years’ development, the database was expanded to represent the interactions of chemical-gene, chemical-disease and gene-disease (Davis et al., 2017). CTD has been suggested as a useful tool to predict potential targets of herbal medicine (Liu et al., 2013; Wang et al., 2017). We searched NAFLD related information in CTD (Supplementary Table S6) and compared with the results from STEM analysis (Figure 3), and then constructed the networks of gene-pathway (Figure 5 and Supplementary Table S7). The network of NALFD progression group consisted of 208 nodes and 346 connections. Of these nodes, 33 nodes were KEGG pathways (15 nodes were overlapping pathways of CTD, 18 nodes were non-overlapping pathways), 91 nodes were DEGs from cluster 11, 41 nodes from cluster 14, 9 nodes from cluster 4 and 34 nodes were other genes from CTD (Figure 5A). A total of 198 nodes and 318 connections were involved in the network of PTFC treatment group. 30 nodes were KEGG pathways in which 18 nodes were overlapping pathways in CTD and 12 nodes were non-overlapping in CTD. 85 nodes were DEGs from cluster 14, 17 from cluster 5, 34 nodes from cluster 11 and other genes were from CTD (Figure 5B).

### Co-expression Gene Modules Identification and Protein-Protein Interaction Networks Construction

**FIGURE 6:**
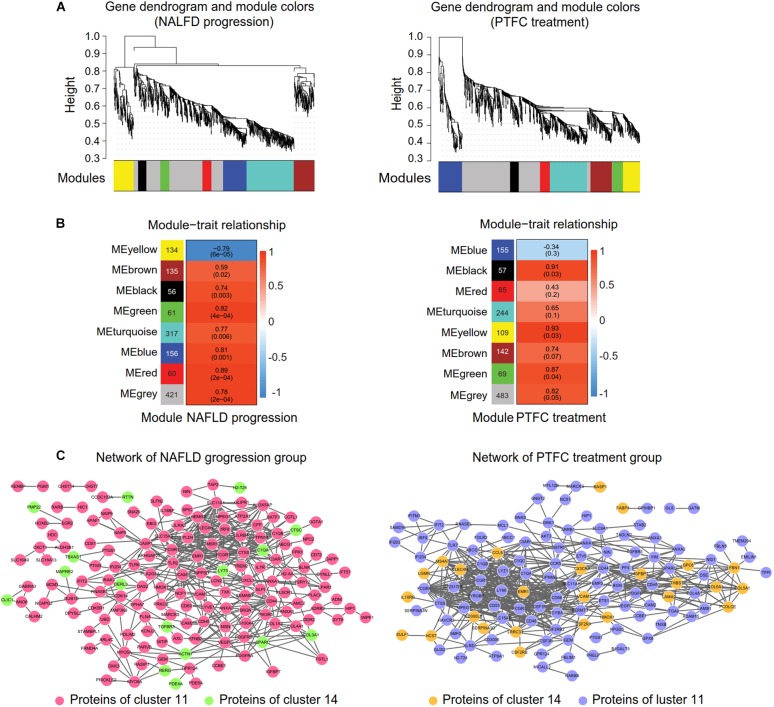
WGCNA and the protein-protein interaction network construction. **(A)** Gene dendrogram and modules colors maps showing modules of co-expressed DEGs of NAFLD progression group (left) and PTFC treatment group (right). Each module is labeled by a unique color (gray module is for DEGs unassigned). Each vertical line in the “leaf” represents a DEG. **(B)** Tables of module-trait relationship of NAFLD progression group (left) or PTFC treatment group (right). Each row corresponds to a module eigengene and each column represents a trait. Each cell reports the correlation coefficient and the *P*-value (in parenthesis) between each module and a trait. The left panel represents eight modules and the number of their member genes. The right panel is a color scale for module trait correlation from –1 to 1. **(C)**. Protein-protein interaction network of NAFLD progression group (left) and of PTFC treatment group (right).

In order to refine the genes that were highly interconnected within NAFLD progression or PTFC treatment, we performed WGCNA (Figure 6) on the clustered DEGs obtained from STEM analysis showed in Figure 3B. WGCNA is a systematic biological approach to build a scale-free network using gene expression data. Genes with highly interconnection will be assigned in a same module via WGCNA (Kogelman and Kadarmideen, 2014). We selected the soft thresholding power 18, 17 (Supplementary Figures S4A,B) and eight gene modules were identified via Dynamic tree cutting (cut height = 0.99), respectively (Figure 6A and Supplementary Table S8). Correlations between modules and NAFLD progression were shown in Figure 6B left (*P*-value 1.2e-87). We selected blocks blue, green and red with higher correlation coefficients than that of the block gray for further analysis (Figure 6B left). With the same way, we selected blocks black, green and yellow for PTFC treatment group (Figure 6A right and Figure 6B right).

Then the protein-protein interaction network of the DEGs in the above selected blocks were constructed via STRING (Supplementary Table S9). The network of NAFLD progression group comprised 163 nodes (145 DEGs from cluster 11 and 18 DEGs from cluster 14) and 437 connections (Figure 6C left). 127 nodes (26 DEGs from cluster 14 and 101 DEGs from cluster 11) and 530 connections were contained in the network of PTFC treatment group (Figure 6C right).

### Screening of the Common Hub Genes Between Networks of NAFLD Progression Group and PTFC Treatment Group

**FIGURE 7:**
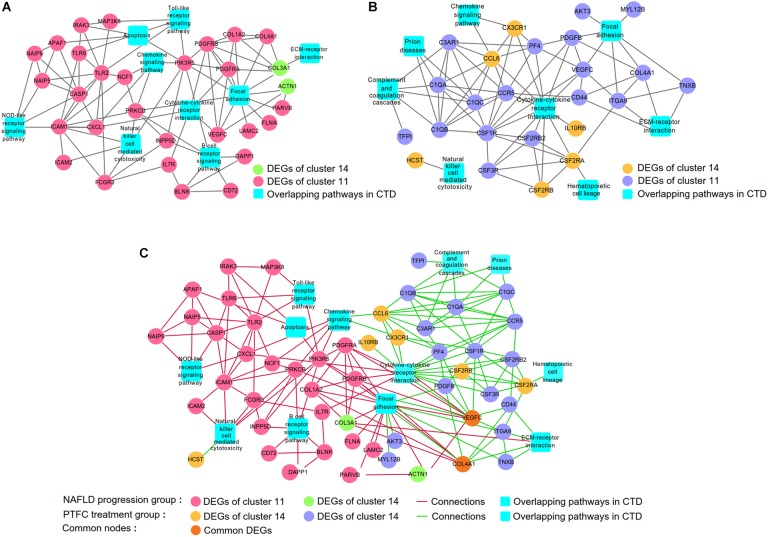
Screening the common hub DEGs between networks of NAFLD progression and PTFC treatment groups. **(A)** Gene-pathway network of NAFLD progression group. **(B)**. Gene-pathway network of PTFC treatment group. **(C)** Merging networks of A and B.

Integrating the analysis based on CTD (Figure 5) and co-expression networks generated by using WGCNA and STRING (Figure 6C), we constructed gene-pathway networks. As shown in Figure 7A, for NALFD progression group, nine pathways were overlapped with CTD, which included Toll-like receptor signaling, Apoptosis, ECM-receptor interaction, Chemokine signaling, Cytokine-cytokine receptor interaction, Focal adhesion, NOD-like receptor signaling, Natural killer cell mediated cytotoxicity and B cell receptor signaling. For PTFC treatment group, eight pathways including of Chemokine signaling, Focal adhesion, Prion diseases, Complement and coagulation cascades, Cytokine-cytokine receptor interaction, ECM-receptor interaction, Natural killer cell mediated cytotoxicity, Hematopoietic cell lineage were overlapped with CTD (Figure 7B).

Comparing the above two networks, we found two common hub genes VEGF-C and COL4A1 which occurred in both networks of NAFLD progression and PTFC treatment groups. In NAFLD progression network, VEGF-C participated in pathways of Cytokine-cytokine receptor interaction and Focal adhesion. COL4A1 participated in Focal adhesion pathway (Figure 7A). In PTFC treatment network, VEGF-C was also involved in Cytokine-cytokine receptor interaction and Focal adhesion pathways. COL4A1 is linked to Focal adhesion pathway (Figure 7B). Merging the two networks (Figure 7C), it clearly showed that both VEGF-C and COL4A1 were involved in networks of NAFLD progression and PTFC treatment and they were screened as the common gene nodes. The result indicated that VEGF-C and COL4A1 play major regulatory roles in the development of NAFLD and might act as targets of PTFC.

### Real-Time PCR and Western Blot Verification

**FIGURE 8:**
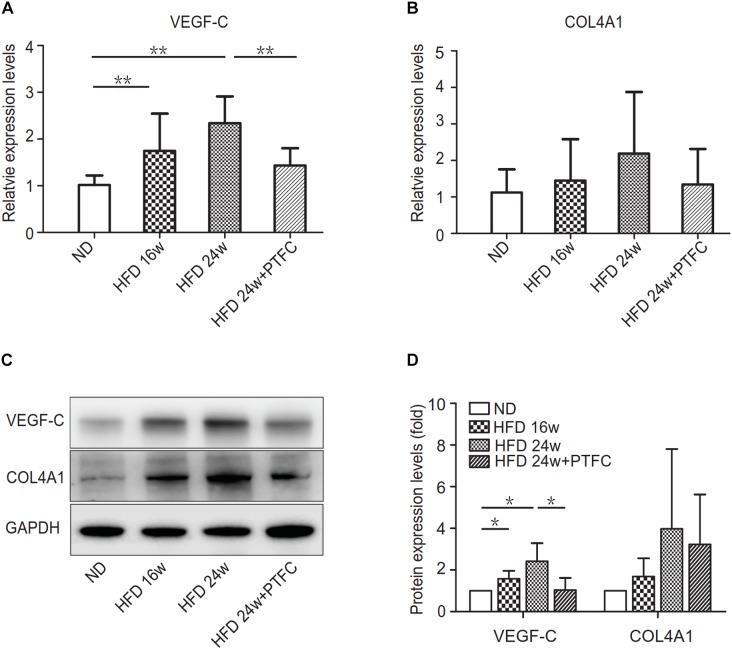
The expression levels of VEGF-C and COL4A1. **(A,B)** Real-time PCR verification of the mRNA expression levels of VEGF-C and COL4A1. Relative transcript levels were calculated via the 2^−ΔΔC(t)^ method and β-actin transcripts were used as the internal control. ^∗^*P* < 0.05, ^∗∗^*P* < 0.01, *n* = 6/group. **(C,D)** Western blot assay of the protein expression levels of VEGF-C and COL4A1. GAPDH was served as the loading control. ^∗^*P* < 0.05.

We conducted real-time PCR and Western blot assay to verify the prediction result. The PCR result showed that during the development of NAFLD, the expression of VEGF-C in liver demonstrated an upward trend, which was significantly different from the control group at 16 and 24 weeks HFD. PTFC treatment significantly decreased the expression of VEGF-C (Figure 8A). Although the expression tendency of COL4A1 is similar to that of VEGF-C, there were no statistically significant differences between the subgroups (Figure 8B). As shown in Figure 8C,D, Western blot assay agreed with the real-time PCR result. VEGF-C and COL4A1 had similar tendencies in protein expression levels, while COL4A1 expression levels in these four subgroups manifested no statistically significant changes.

## Discussion

Natural products or Chinese herbal medicine are valuable resources for NALFD treatment. Network pharmacology is an efficient strategy to predict the potential targets of the natural products or Chinese herbal medicine. We previously reported that PTFC effectively reduced the symptoms of NAFLD (Chen et al., 2014; Wu et al., 2017). To understand the functional mechanism of PTFC, it is necessary to identify the gene targets of PTFC. In the present study, we conducted data mining and predicted the potential molecular targets of PTFC via network pharmacology using the microarray data of our previous report (Wu et al., 2017). Currently, the potential targets that predicted by network pharmacology include many molecules, making it difficult to choose from them for further in-depth research. Considering the relationship between the progression of NAFLD and PTFC treatment, we classified the raw data into NAFLD progression group and PTFC treatment group. Then we conducted a parallel analysis on these two data sets and tried to find the overlapping genes as the candidate targets genes. In addition, during the data analysis process, the data were filtered via the CTD database to ensure that the information obtained is only related to NAFLD.

We first obtained the networks of the NAFLD progression group and the PTFC treatment group (Figure 7A,B), respectively. The Toll-like receptor signaling pathway and Chemokine Signaling pathway were found in the NAFLD progression network (Figure 7A). We previously reported the alteration of the members of these two pathways with PTFC treatment (Wu et al., 2017). The result of this study further confirmed these two pathways played important roles in the progression of NAFLD. In addition, Chemokine pathway was identified in PTFC network, suggesting that this pathway exerted important effects in PTFC treatment. These results also indicated that the method adopted in this study was feasible.

**FIGURE 9:**
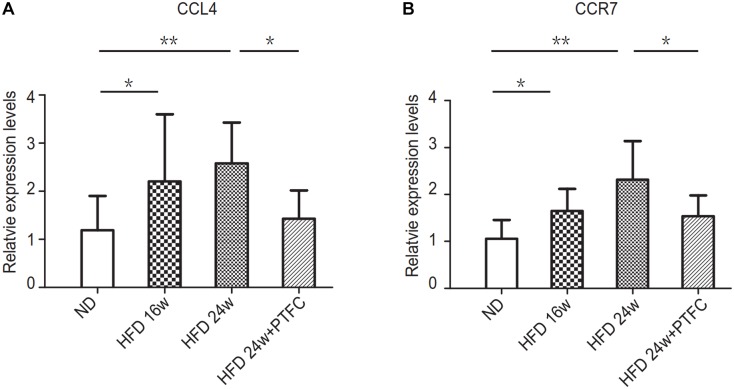
The transcript expression levels of CCL4 and CCR7. Real-time PCR verification of the mRNA expression levels of CCL4 and CCR7. Relative transcript levels were calculated via the 2^−ΔΔC(t)^ method and β-actin transcripts were used as the internal control. ^∗^*P* < 0.05, ^∗∗^*P* < 0.01, *n* = 6/group.

The real-time PCR and Western blot verifications revealed that there was statistically significant difference in the expression levels of VEGF-C between ND and 16 or 24 weeks HFD or between the subgroups of 24-week HFD and PTFC treatment. While there were no statistically significant differences in the expression levels of COL4A1 among subgroups. Therefore, we selected VEGF-C as the key target of PTFC against NAFLD. In order to confirm this result, we detected the mRNA expression levels of CCL4 and CCR7, the upstream regulated genes of VEGF-C (Yu et al., 2017; Lien et al., 2018) by real-time PCR. The results revealed that the expression levels of CCL4 and CCR7 in 16-week HFD and 24-week HFD subgroups were significantly increased compared with that of ND subgroup. PTFC treatment significantly reduced their expression levels (Figure 9). This result indicated that the predicted target VEGF-C and its upstream regulated genes have similar expression tendencies in the four subgroups.

Vascular endothelial growth factor-C is a member of the VEGF superfamily, which is critical for angiogenesis and lymphangiogenesis (McColl et al., 2004). VEGF-C is the main regulator for lymphangiogenesis and functions mainly through its receptor VEGF receptor 3 (VEGFR-3) (Rauniyar et al., 2018). The studies showed that VEGF-C plays important role in tumor metastasis. Therefore, VEGF-C and its receptor VEGFR-3 have been considered as the promising therapeutic target in cancer (Yamakawa et al., 2018). Intriguingly, VEGF-C was found a potential regulator for dietary regulation of adiposity and cholesterol metabolism because it is required for intestinal lymphatic vessel maintenance and fat absorption (Nurmi et al., 2015). Clinical investigation demonstrated that serum VEGF-C and VEGF-A levels are higher in obese subjects and VEGF-C rather than VEGF-A is closely related to dyslipidemia and atherosclerosis (Wada et al., 2011). VEGF-C overexpression mice have increased weight gain and ectopic lipid accumulation, showing the phenotype of insulin resistant and enhanced pro-inflammatory on normal chow (Karaman et al., 2016). Transgenic mice that constitutively express soluble-VEGFR-3-lg show reduced inflammation in adipose tissue, decreased hepatic lipid accumulation and improved metabolic parameters than control group under HFD (Karaman et al., 2015). It was also reported that VEGF-C functions in myofibroblast differentiation, proliferation and migration and increase fibrosis by activation transforming growth factor (TGF)-β and ERK pathways (Zhao et al., 2014). These findings indicated that, in addition to lymphangiogenesis, VEGF-C plays roles in lipid accumulation, obesity-related insulin resistant, inflammation as well as in fibrosis, which are also important characteristics in the development of NAFLD. PTFC has the effects of lipid reducing (Chen et al., 2014; Yang et al., 2017), anti-inflammation (Chen et al., 2014; Wu et al., 2017), anti-fibrosis (Wu et al., 2017). Hesperidin, one of the main component of PTFC, has been reported to inhibit obesity and attenuates insulin resistance (Pu, 2016). Therefore, we speculate that VEGF-C as the therapeutic target of PTFC may improve hepatic lipid accumulation, insulin resistant, inflammation and fibrosis.

**FIGURE 10:**
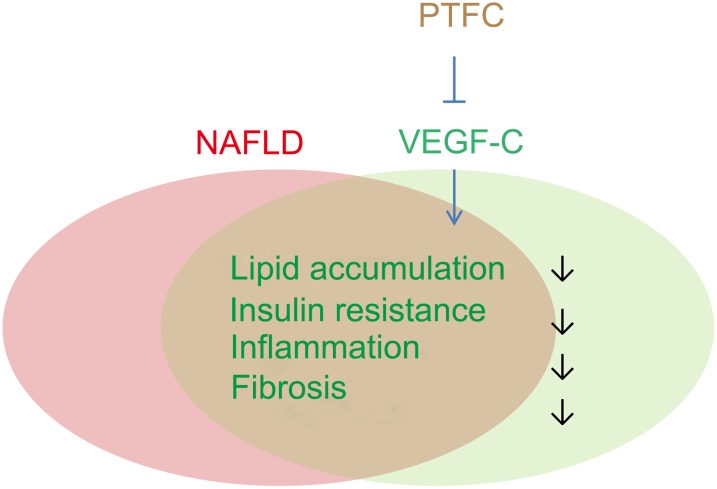
Potential roles of VEGF-C in the progression of NAFLD and PTFC treatment. VEGF-C plays roles in lipid accumulation, obesity-related insulin resistant, inflammation as well as in fibrosis, which are also important characteristics in the progression of NAFLD. The effects of lipid reducing, anti-inflammation, anti-fibrosis and attenuation of insulin resistance of PTFC may be associated with targeting VEGF-C.

Although network pharmacology has its limitations and cannot replace biological functional verification, our results strongly suggest that VEGF-C is a key target of PTFC against NAFLD. The effects of lipid reducing, anti-inflammation, anti-fibrosis and attenuation of insulin resistance of PTFC may be closely related to the reduction of VEGF-C (Figure 10). Our findings also indicate that VEGF-C might be an effective target for developing effective therapeutic strategies against NAFLD. So far, the roles of VEGF-C in the progression of NAFLD and in the PTFC treatment have not been directly described. Further investigations need to be conducted with liver-specific VEGF-C knockout mice.

## Ethics Statement

This study was carried out in accordance with the recommendations of international, national, and/or institutional guidelines for the care. The protocol was approved by The First Affiliated Hospital of Zhejiang Chinese Medical University.

## Author Contributions

ZC initiated the project, designed the experiments, and revised the manuscript. WH analyzed the data and wrote the manuscript. SL performed the real-time PCR and Western blot. LW, BH, and JJ performed the animal experiments. All authors read and approved the final manuscript.

## Conflict of Interest Statement

The authors declare that the research was conducted in the absence of any commercial or financial relationships that could be construed as a potential conflict of interest.

## Footnotes

1 https://www.ncbi.nlm.nih.gov/geo/query/acc.cgi?acc=GSE128850

2 http://ctd.mdibl.org/

3 http://string-db.org

4 http://expressconverter.software.informer.com/

5 http://bioconductor.org/packages/release/bioc/html/limma.html

6 https://cran.r-project.org/web/packages/psych/index.html

7 https://cran.r-project.org/web/packages/pheatmap/index.html

8 https://david.ncifcrf.gov/

9 http://www.cytoscape.org/

10 https://cran.r-project.org/web/packages/VennDiagram/index.html

11 http://www.cs.cmu.edu/jernst/stem/

12 https://cran.r-project.org/web/packages/WGCNA/

13 https://string-db.org/

14 http://ctdbase.org/
