# Supplementary material for: Prediction of VEGF-C as a Key Target of Pure Total Flavonoids From Citrus Against NAFLD in Mice via Network Pharmacology
Source: Front Pharmacol. 2019 Jun 4;10:582. doi: 10.3389/fphar.2019.00582 (PMC6558193; doi:10.3389/fphar.2019.00582)
Supplement: Supplementary file 10 [file Data_Sheet_1.docx]

Supplementary Material


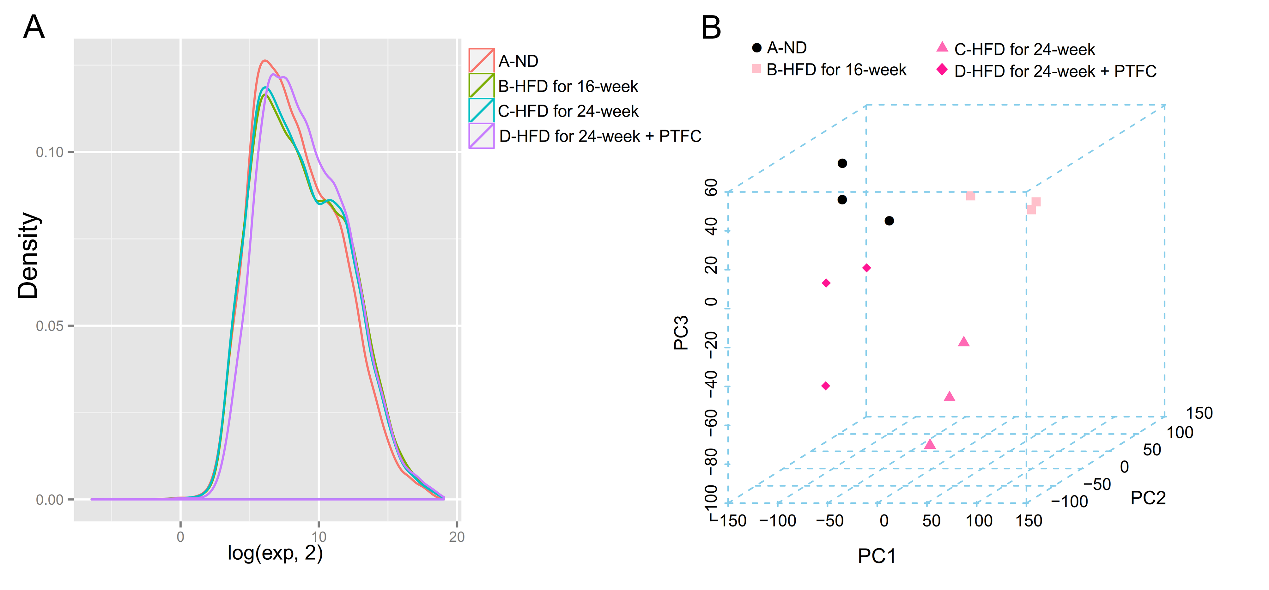


**Supplementary Figure 1**. Data quality control. (A). Density distribution of the normalized gene expression profiles. (B). Principal component analysis for all normalized transcripts.


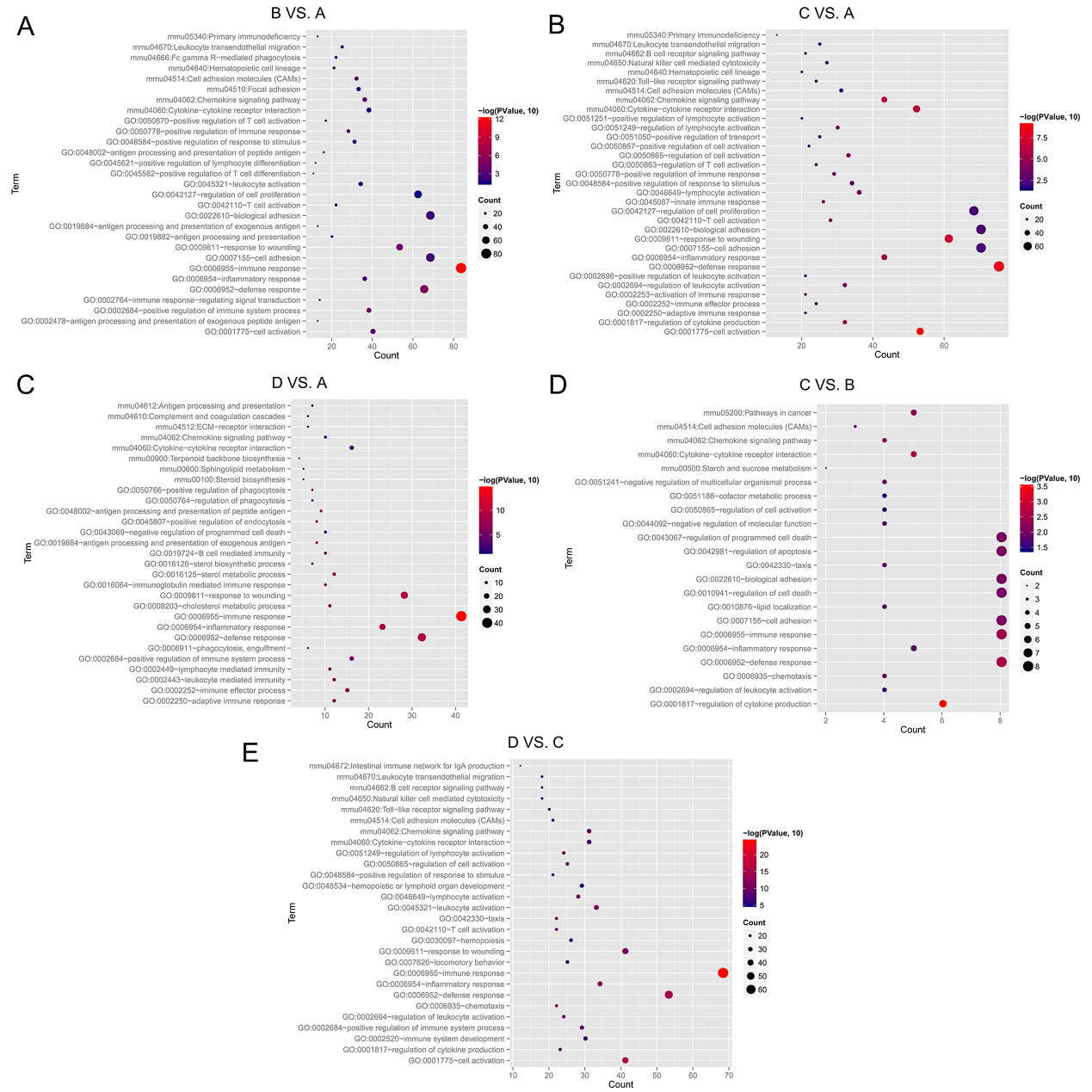


**Supplementary Figure 2**. Gene Ontology (GO) Biological Process and Kyoto Encyclopedia of Genes and Genomes (KEGG) pathway enrichment analysis of DEGs in five comparisons. The vertical axis represents the numbers of DEGs in a particular GO or KEGG term. The size of the dots represents the number of the DEGs and the color donates the range of P values. (A-E) Comparisons between subgroups B and

A, C and A, D and A, C and B, D and C.


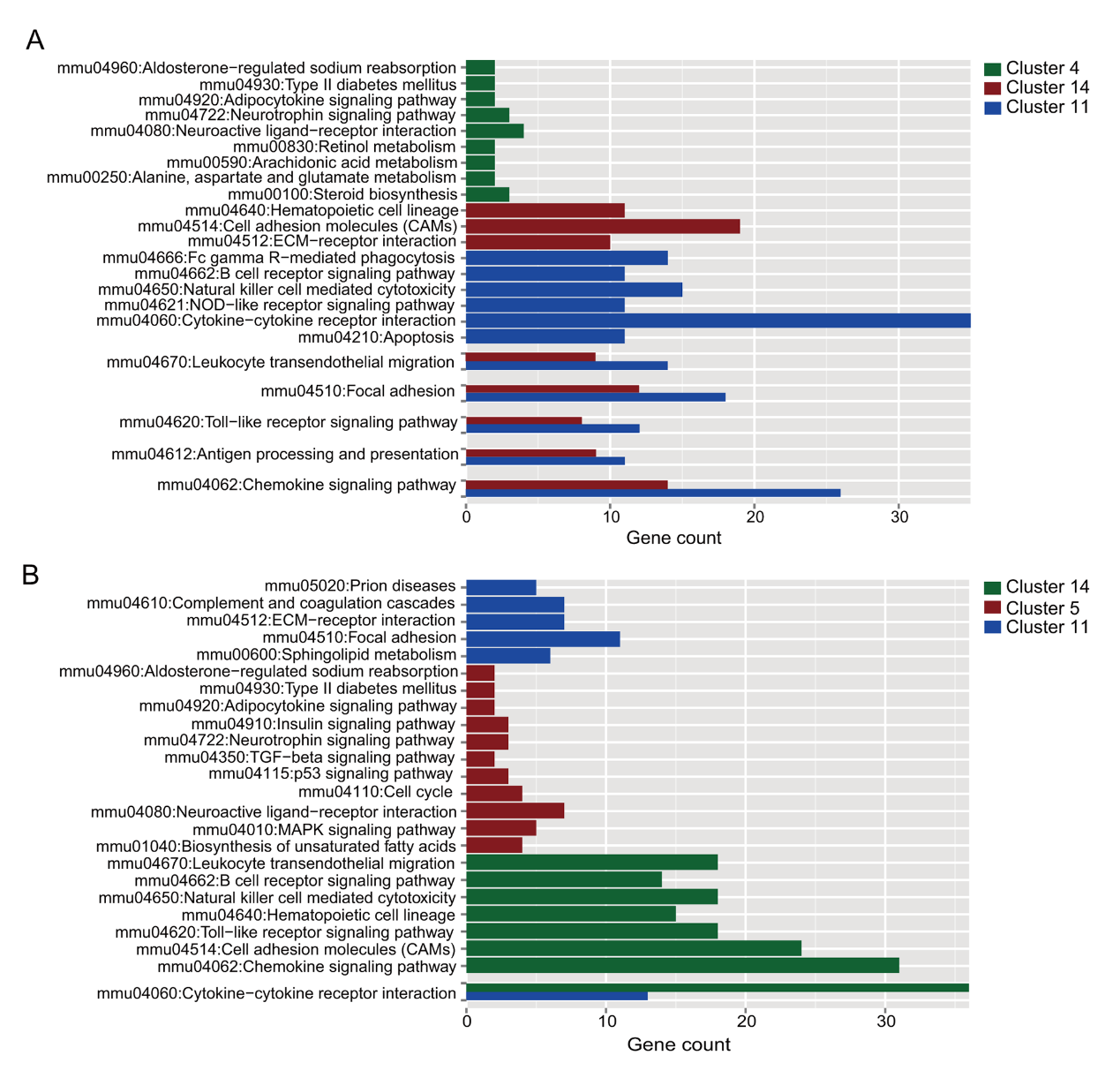


**Supplementary Figure 3**. The KEGG pathway enrichment analyses of DEGs in the STEM clusters. (A).KEGG pathway enrichment of NAFLD progression group. (B). KEGG pathway enrichment of PTFC treatment group.


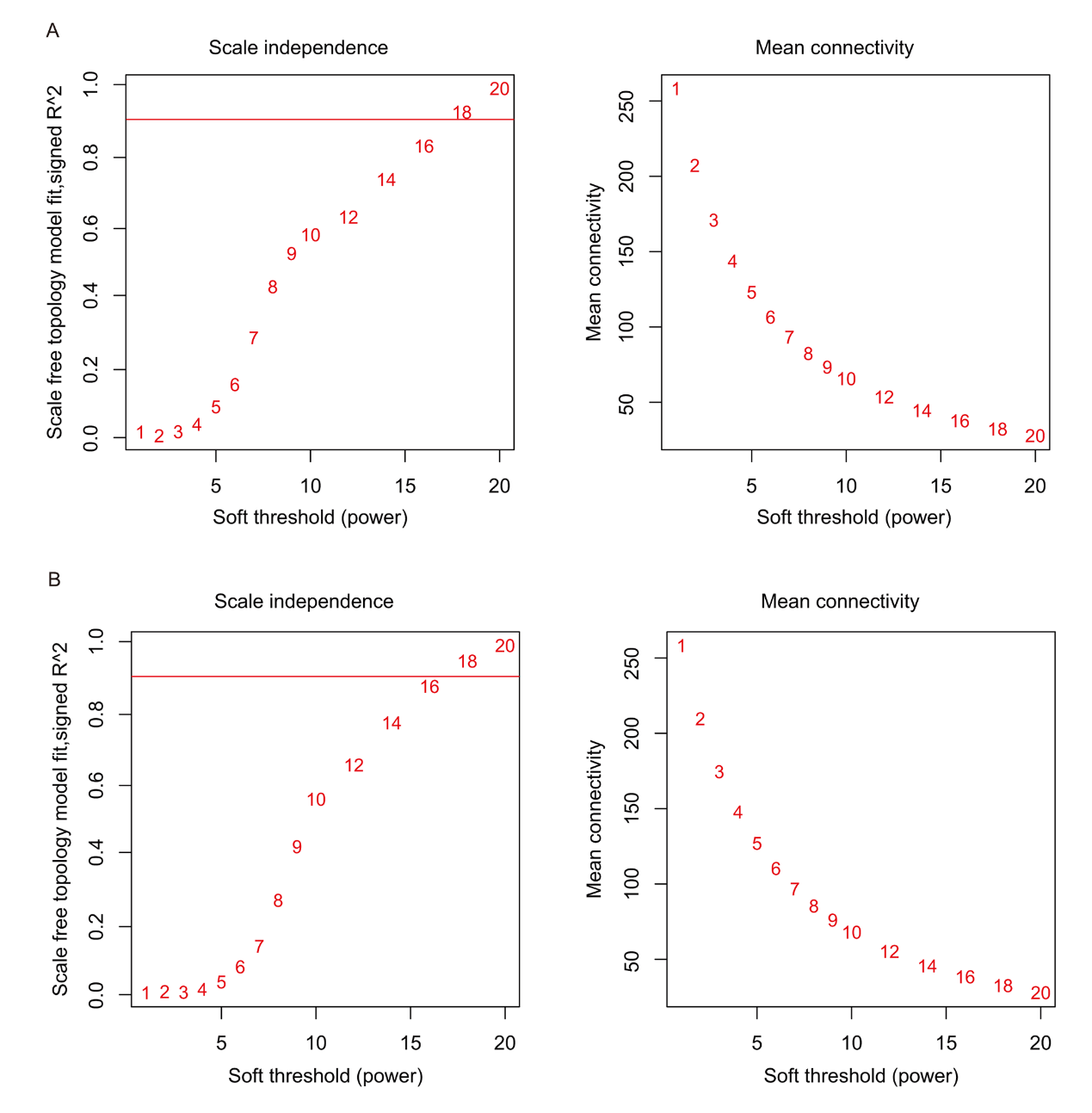


**Supplementary Figure 4.** The soft-thresholding power calculation of WGCNA analysis. (A). The Soft-thresholding power calculation of NAFLD progression group. (B). The Soft-thresholding power calculation of PTFC treatment group. The left panel shows scale independence. The right panel represents mean connectivity.

**Supplemental tables:**

**Table S1.** The expression levels of DEGs in the five pairwise comparisons of B vs A, C vs A, D vs A, C vs B and D vs C.

**Table S2**. GO biological process and KEGG pathway enrichment analyses of DEGs in the five pairwise comparisons of B vs A, C vs A, D vs A, C vs B and D vs C.

**Table S3**. Analysis of sets of differentially expressed genes of NAFLD progression group and PTFC treatment group.

**Table S4**. DEGs in each STEM cluster of NAFLD progression group and PTFC treatment group.

**Table S5.** The KEGG pathway enrichment analysis of the DEGs from significant STEM clusters.

**Table S6**. NAFLD related information in CTD.

**Table S7.** Information of gene-pathway networks of NAFLD progression group and PTFC treatment group.

**Table S8.** Information of gene modules of co-expressed DEGs.

**Table S9.** Protein-protein interaction networks construction based on STRING.
